# Homeostasis of lymphocytes and monocytes in frequent blood donors

**DOI:** 10.3389/fimmu.2012.00271

**Published:** 2012-08-22

**Authors:** Robert B. Ratts, Nan-Ping Weng

**Affiliations:** Laboratory of Molecular Biology and Immunology, National Institute on Aging, National Institutes of HealthBaltimore, MD, USA

**Keywords:** T cell, telomere, telomerase aging, leukapheresis, TREC, CD31

## Abstract

Age-associated decline of immune function is believed to be mainly due to alterations of immune cells. However, longitudinal changes of human immune cells with age have not yet been adequately addressed. To test the hypothesis that regeneration of lymphocytes and monocytes is robust throughout most of adult life until advanced age, we examined six leukapheresis donors (3 young and 3 middle-aged/old) who donated approximately 10% of their peripheral blood mononuclear cells (PBMC) every other month over 3–5 years. We found the number of both lymphocytes and monocytes were quite stable in the blood of all six donors. As expected, young donors had more T cell receptor excision circles (TRECs), CD31{}^+^ cells (CD4 only) and longer telomeres in T cells than did the middle-aged donors. Interestingly, more variation in TREC number, Vβ usages, and telomere lengths were observed in young donors during the 3–5 years course of donation whereas the middle-aged/old donors showed a rather striking stability in these measurements. This may reflect a more prominent role of thymic output in T cell regeneration in young than in middle-aged/old donors. Together, these findings provide an *in vivo* glimpse into the homeostasis of lymphocytes and monocytes in the blood at different ages, and support the notion that regeneration of lymphocytes and monocytes is robust throughout adult life up to the early 70s.

## INTRODUCTION

Homeostasis plays an essential role in the maintenance of normal immune function. For most of adult life, a dedicated balance is conserved between the production of new immune cells and the removal of old or dysfunctional cells and stable cell numbers and function are sustained. However, this balance is believed to be altered in the later stages of life which results in immune function decline in the elderly. Cross-sectional studies of human age-associated changes have generated a wealth of information and provide a foundation for our current understanding of immune system aging (Douek et al., 1998; Weng, 2006; Goronzy et al., 2007). Due to the lack of similar longitudinal studies, the temporal order of immune function changes *in vivo* with age as well as the relative contributions of aging and other factors such as generational differences or environmental changes over time are not fully understood.

T cell precursors initiate development in the bone marrow and subsequently migrate to the thymus where they undergo a series of tightly regulated differentiation processes to become mature naïve T cells. First, genomic DNA is rearranged at the TCR gene loci to produce T cells each with a unique TCR sequence combination. During this recombination process one variable β gene is joined with a “D” and a “Jβ” segment to form a functional variable β (Vβ) chain and this is followed by the rearrangement of one variable α (Vα) gene with a Jα gene segment. Together, they form a functional TCR. Second, T cells with functionally rearranged TCR α and β chains undergo positive and negative selection to become mature T cells and subsequently migrate to the peripheral lymphoid organs and blood. The output of thymocytes has been estimated by the amount of T cell receptor excision circles (TREC), generated from the TCR gene rearrangement, in the T cell population (Douek et al., 1998). Conditions of high thymic export such as found in children are associated with high TREC levels whereas the opposite conditions such as found in the elderly display low TREC levels (Douek et al., 1998; Harris et al., 2005; Naylor et al., 2005; van den Dool and de Boer, 2006). CD31, a cell adhesion molecule, is expressed on a subset of naïve CD4 T cells containing high TREC levels. The percentage of CD31^+^ CD4 T cells decreases with age and thus CD31^+^ CD4 T cells are considered as recent thymic emigrants (Kimmig et al., 2002).

In addition to thymic production, naïve T cells can be expanded by homeostatic proliferation in the periphery. According to an estimate by mathematical modeling of normal young adults, thymic output accounts for approximately one-third of the naïve T cells and peripheral homeostatic proliferation generates about two-thirds of naïve T cells (Bains et al., 2009). Cytokines such as IL-7 and IL-15 are essential to the peripheral homeostatic proliferation of T cells (Surh et al., 2006) and the overall diversity of T cell repertoire does not appear to be altered by this process until late in life (Naylor et al., 2005). The age-associated reduction of TCR repertoire is a result of reduced production of naïve T cells and the biased expansion of some T cell clones (Naylor et al., 2005). Whether the uneven expansion of some T cell clones is due to antigen-driven selection and/or “dysfunction” of homeostatic proliferation with age requires further study.

Telomeres, the termini of chromosomes, are essential for chromosomal integrity (Blackburn, 2005; de Lange, 2005). Attrition of telomere length occurs due to the inability of conventional DNA polymerases to completely replicate telomeres during chromosomal replication. Our previous studies have shown naïve CD4 T cells possess longer telomeres than do memory CD4 T cells (Weng et al., 1995), and telomere shortening occurs in both the CD4 and CD8 T cell compartments with age (Son et al., 2000). Thus, telomere length has been viewed as an indicator of previous cell division history and a predictor for the residual replicative life span of a cell. Telomerase is an enzyme that synthesizes telomeres and compensates for telomere loss during cell division. Telomere length in lymphocytes is a consequence of the interplay of telomerase-mediated telomere synthesis and DNA replication-associated telomere loss. Lymphocytes express telomerase in a tightly regulated fashion during their development and activation (Weng et al., 1996). Resting T cells express low to undetectable levels of telomerase whereas engagement of TCR or stimulation by homeostatic cytokines induces telomerase activity. When telomerase activity is highly induced, telomere length appears to be maintained in these actively dividing T cells (Weng et al., 1997; Li et al., 2005).

Previous studies of lymphocyte regeneration *in vivo* have been mainly focused on abnormal conditions (Sachsenberg et al., 1998; McCune et al., 2000; Neese et al., 2002; Douek et al., 2003; Krupica et al., 2006; Williams et al., 2007; Daguindau et al., 2008). *In vivo* lymphocyte homeostasis and the influence of age on this process have not been extensively analyzed in normal adults. Here, we report an analysis of six frequent leukapheresis donors (3 young donors with average starting age of 25 and 3 middle-aged/old donors with an average starting age of 61) who donated approximately 10% of peripheral blood mononuclear cells (PBMC) every other month continuously over 3–5 years. The older donors span a large age range (50–73), they are referred to herein as “middle-aged/old” group. We found that the numbers of both lymphocytes and monoctyes were quite stable in blood for all six donors over the course of donation, and that the young donors had more TRECs, and longer telomeres in both CD4 and CD8 T cells than the middle-aged/old donors. In addition, young donors also had more CD31^+^ naïve CD4 T cells than did the middle-aged/old donors. Unexpectedly, young donors exhibited a great deal of variation or fluctuation in TREC levels, Vβ usages, and telomere lengths when compared with more stable measurements observed in the middle-aged/old donors. Together, our findings provide new information on the homeostasis of blood lymphocytes and monocyte *in vivo* in young and middle-aged/old humans and suggest the regeneration of lymphocytes and monocytes is robust throughout adult life up to the early 70s.

## MATERIALS AND METHODS

### COLLECTION OF PBMC BY LEUKAPHERESIS OF NORMAL DONORS

Six normal volunteers 22–70 years old were leukapheresed at the Clinical Research Branch of the National Institute on Aging under an Institutional Review Board-approved protocol. Informed consent was obtained from all subjects. PBMC were further isolated using Ficoll gradient centrifugation and cryopreserved for 1–5 years for subsequent analysis.

### ISOLATION OF T AND B CELLS FROM PBMC

The procedures for isolating T and B cells from PBMC were previously described (Son et al., 2000). In brief, cryopreserved PBMC were thawed slowly and washed with Hank’s balanced salts solution (HBSS) containing 0.2% bovine serum albumin (BSA), 0.01 M HEPES, and 50 U/ml penicillin and 50 μg/ml streptomycin. CD4 and CD8 T cells, and B cells (CD19^+^) were isolated by positive immunomagnetic separation using Dynabeads (Invitrogen, Life Science) according to the manufacture’s directions. Purities of isolated T and B cells were typically >95%.

### FACS ANALYSIS OF LYMPHOCYTE MARKERS

Thawed PBMC were washed twice with HBSS containing 0.2% BSA and 0.1% NaN_3_ and stained with the following antibodies in different combinations. Fluorescein isothiocyanate (FITC)-labeled antibodies against CD31, phycoerythrin (PE)-labeled CD62L, CCR7, CD28, CD127, PE-Cy5-labeled CD45RA, CD8, PE-Cy5.5-labeled CD4; and allophycocyanin (APC) labeled CD8, CD4, and CD31 were purchased from eBioscience (San Diego, CA, USA). FITC-labeled CD16, CD25, CD56, PE-Cy5-labeled CD3, and APC-labeled CD19 were purchased from Invitrogen. Cells were stained according to the manufacture’s instructions. Stained cells were run on a Calibur flow cytometer or FACS Canto II flow cytometer (BD Biosciences, San Jose, CA, USA), and the data were further analyzed by CellQuest Pro (BD Biosciences) v5.2.1 or FlowJo v8.8.6 respectively (TreeStar Inc., Ashland, OR, USA).

### TCR Vβ REPERTOIRE ANALYSIS BY FACS

TCR Vβ repertoire usage was analyzed using a panel of fluorochrome-conjugated antibodies against 24 different Vβ based on the manufacturer’s instructions (Beckman Coulter, Sykesville, MD). PBMC were stained with three different Vβ antibodies plus antibodies to CD4 and CD8. Data were collected as described above. Each Vβ was examined at three to five distinct times spanning the largest interval possible for each donor. The percentages of each Vβ in CD4 and CD8 T cells were determined by CellQuest Pro or FlowJo v8.8.6. Samples were normalized to compensate for the fact that some had more time points than others. Fluctuations due to logistic and technical issues were identified by measuring the same Vβ two to three different times from the same donors at the same time points. The SD of the mean of each Vβ from all measurements of six donors was calculated.

### TREC ANALYSIS

TREC analysis was previously described (Douek et al., 2000). In brief, genomic DNA was purified from CD4 and CD8 T cells using a Gentra Puregene Cell Kit according to the manufacturer’s instructions (Gentra Systems, Inc., Minneapolis, MN,USA). The frequency of the δRec-ψJα TREC created when the TCRD segment is excised from the TCR β gene locus was determined using Taqman real-time PCR 7500 Fast Real-Time PCR System (Applied Biosystems). A TREC standard was used for quantitation (gift from Dr. Daniel C. Douek, National Institute of Allergy and Infectious Diseases, NIH) as previously described (Douek et al., 2000). DNA levels were determined using a NanoDrop ND-1000 spectrophometer (NanoDrop Technologies, Wilmington, DE, USA) and further normalized using the house keeping gene RPL-32. TRECs per μg were converted to TREC per cell based on the DNA content of 1.5 million cells containing 1 μg of DNA (Bains et al., 2009).

### TELOMERE LENGTH ANALYSIS BY FLOW FISH

Determination of telomere lengths by flow FISH was previously described (Rufer et al., 1998). Briefly, isolated CD4 and CD8 T cells were permeabilized using cytofix cytoperm (BD Biosciences), and hybridized with telomere-specific FITC-labeled PNA probes (Perkin Elmer, Walthan, MA, USA) in 70% deionized formamide (Invitrogen) overnight. Cells were stained with 7AAD (BD biosciences). Data were collected on a linear scale on FL1 channel, and the mean florescence intensity was determined using CellQuest Pro software. Mean florescence intensity was normalized using beads (Bangs Laboratories, Inc., Fishers, IN,USA) and then converted to kilobases using an equation derived from a series of samples whose telomere length was measured by both Southern blot and Flow-FISH.

### STATISTICAL ANALYSIS

Error bars represent the SD. *P*-values are the result of a Student’s *t*-tests and *P* < 0.05 was considered to be statistically significant.

## RESULTS

### STABLE LYMPHOCYTE AND MONOCYTE COUNTS IN FREQUENT LEUKAPHERESIS DONORS OVER 3–5 YEARS

**FIGURE 1:**
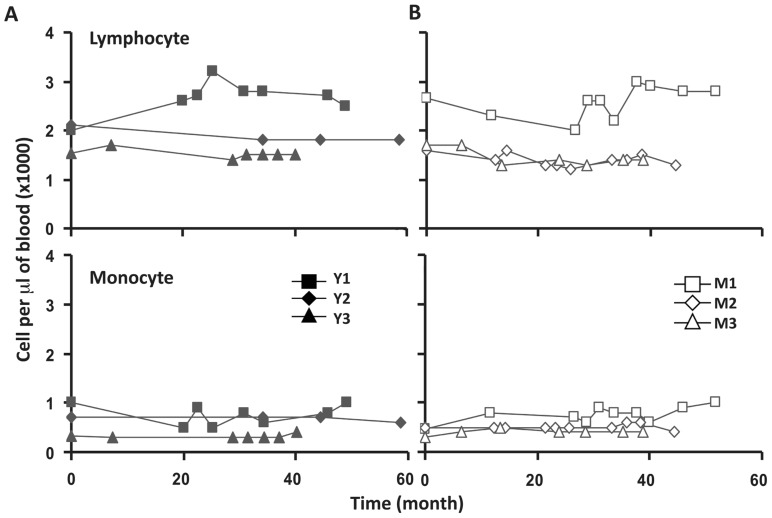
**Lymphocyte and monocytes counts were stable in peripheral blood of six frequent leukapheresis donors throughout 3–5 years.** Six normal donors described in **Table 1** were leukapheresed every other month over the course of 3–5 years. Lymphocyte (top panels) and monocyte (bottom panels) counts in peripheral blood were determined as part of a complete blood count test. Cell samples were collected at different times from each donor. For simplicity, 0 on the abscissa was the first sample collected from each donor which does not necessarily denote the first donation. Young donors are shown on the left and middle-aged donors on the right.

**FIGURE 2:**
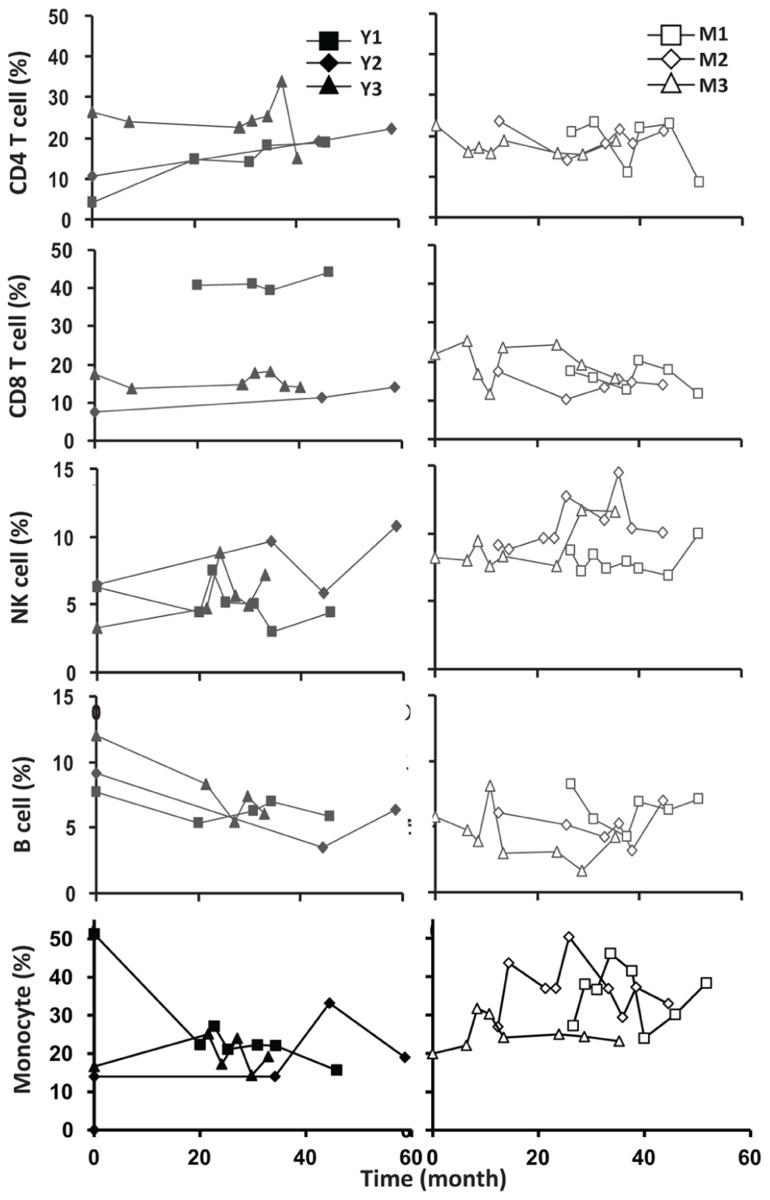
**Frequent luekapeheresis did not alter frequency of PBMC constituents.** Donors underwent leukapheresis every other month and the relative percentages of componen T cell types were determined from PBMC by FACS analysis. Young donors (left panels) were compared with middle-aged donors (right panels). Starting from the top CD4, CD8, NK cell (CD16/CD56^+^), B cells (CD19^+^), and monocyte (CD14^+^) were examined. Time values on the abscissa correspond to the time values on the abscissa in **Figure 1**.

**Figure A1:**
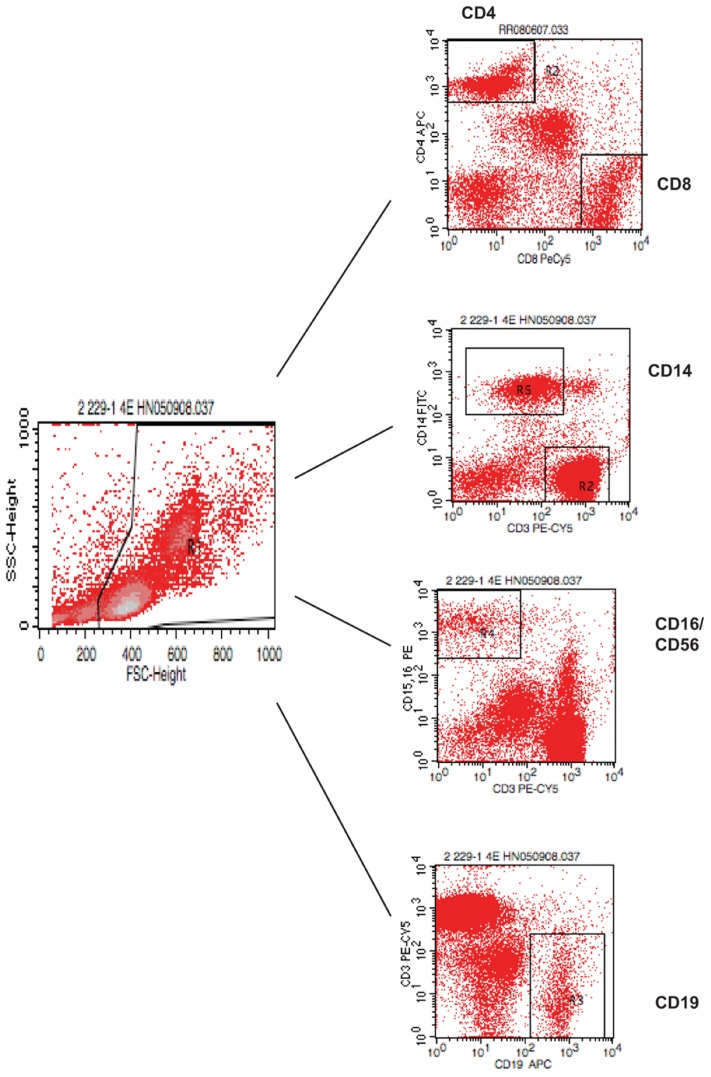
**Gating strategy for flow cytometry analysis of blood lymphocytes.** PBMC were isolated and stained with various antibodies and analyzed by FACS.

To assess the homeostasis of blood leukocytes in healthy human adults, we examined the cell counts of lymphocytes and monocytes of six donors who underwent frequent (every 2–4 months over a period of 3–5 years) leukapheresis (**Table 1**). In view of the effect of age, we divided these six donors into two age groups: 3 young (mean starting age 25 ± 3) and 3 middle-aged/old (mean starting age 61 ± 10). Lymphocyte and monocyte counts were determined by CBC and are presented in **Figure 1**. Some fluctuations were found, but the overall cell counts for both lymphocytes and monocytes were stable. We then focused on the subpopulations of lymphocytes, including CD4 and CD8 T cells, B cells (CD19^+^), and NK cells (CD16^+^/CD56^+^) by flow cytometry analysis (Gating strategy in **Figure A1** in Appendix). The number of CD4 and CD8 T cells and monocytes appeared stable. In contrast, NK cells exhibited a small increase in middle-aged donors while a decrease of B cells was observed in young donors (**Figure 2**). Together, these findings suggest that the numbers of lymphocytes and monocytes were well maintained in these frequent blood donors over 3–5 years time regardless of their age.

**Table 1:** Subject age, gender, and donation number.

Alias^a^	Gender	Age range	Donation#^b^
Y1	M	24-27	17 (7)
Y2	M	22-27	18 (4)
Y3	F	28-32	13 (7)
M1	M	50-54	22 (10)
M2	M	62-66	17 (10)
M3	F	70-73	13 (6)

*^a^Normal subjects underwent leukapheresis over 3–5 years once every 2–4 months. Young (Y) subjects with the mean starting age = 25 and middle/oldaged (M) with mean age = 61.*

*^b^The number of donations in NIA clinic covered in this study. Not all time point over this span were examined.The number in parenthesis indicated times points examined for each donor.*

### VARIED THYMIC OUTPUT IN YOUNG AND IN MIDDLE-AGED/OLD ADULTS

**FIGURE 3:**
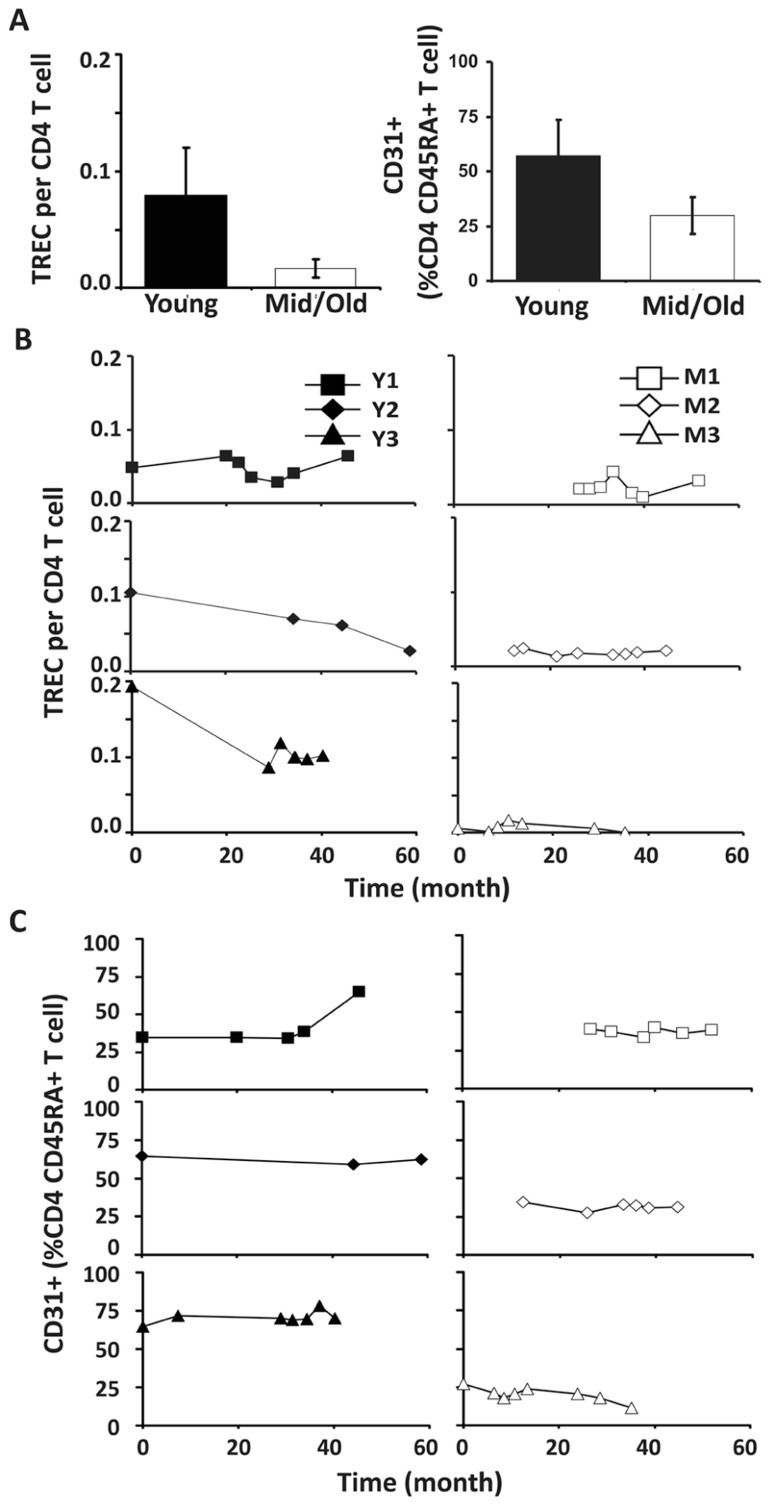
**Decreased thymic CD4 output was observed in young but not middle-aged donors.** **(A)** TREC counts (left) and CD31^+^ percentages in naïve CD4 T cells (right) of young and middle-aged donors. PBMC were isolated from leukapheresis blood and CD4 T cells were further isolated via positive selection. TREC content was examined longitudinally by Taqman Real time PCR. CD31 expression was determined by flow cytometry analysis. The average of all time points were presented with the SD (*P* ≤ 0.05). **(B)** TREC frequencies in CD4 T cells isolated from young (left panels) and middle-aged (right panels) donors are shown. **(C)** CD31^+^CD45RA^+^CD4 T cells.

To analyze the relative contribution of thymic output in young and middle-aged/old adults, we examined TREC levels in CD4 and CD8 T cells in our donors. In agreement with the previous findings (Douek et al., 1998; Harris et al., 2005; Naylor et al., 2005; van den Dool and de Boer, 2006), TREC counts in CD4 T cells were higher in young than in middle-aged/old donors (*p* < 0.05; **Figure 3A**). Interestingly, we observed three different patterns in young donors: no obvious change, a decrease during the first 2 years but stable after that, and a gradual decline (**Figure 3B**). In contrast, TREC levels were low but stable in the middle-aged/old donors over similar lengths of time (**Figure 3B**). This suggests that the thymic output in young is more variable than in middle-aged/old adults.

**Figure A2:**
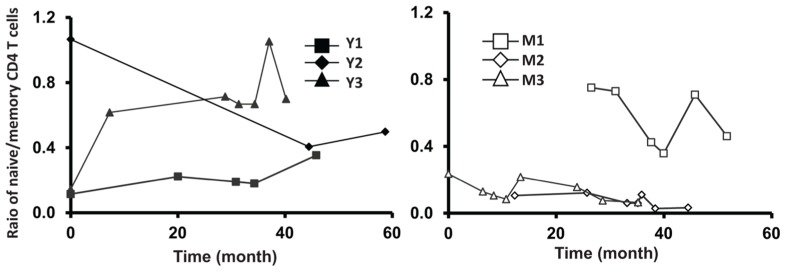
**Relative stable ratio of naïve/memory CD4 T cells of six frequent leukapheresis donors throughout 3–5 years.** PBMC were isolated and stained with CD4, CD45RA, and CCR7. naïve CD4 T cells were defined by CD4^+^CD45RA^+^CCR7^+^ and memory cells were defined by CD4^+^CD45RA^-^CCR7^+/-^

Next, we analyzed the percentage of CD31^+^ T cells in the CD4^+^CD45RA^+^ T cell compartment. Similar to TREC counts, we observed fewer CD31^+^ T cells in the CD4^+^CD45RA^+^ compartment in middle-aged/old adults than in young donors (*P* < 0.05; **Figure 3C**). Furthermore, the percentage of CD31^+^ CD45RA^+^CD4 naïve T cells was stable in middle-aged/old donors (**Figure 3C**). Interestingly, the percentage of CD31^+^ CD45RA^+^CD4 naïve T cells was also stable in young donors, even though some displayed decreasing TREC frequencies (particularly Y3). This suggests TREC levels may be more sensitive than CD31 expression in measuring thymic output. We further analyzed the ratios of naïve/memory T cells over the course of leukapheresis and found that there were individual differences in the ratios of naïve/memory T cells but did not observe a clear cut age-related difference between young and middle-aged/old donors (**Figure A2** in Appendix).

**FIGURE 4:**
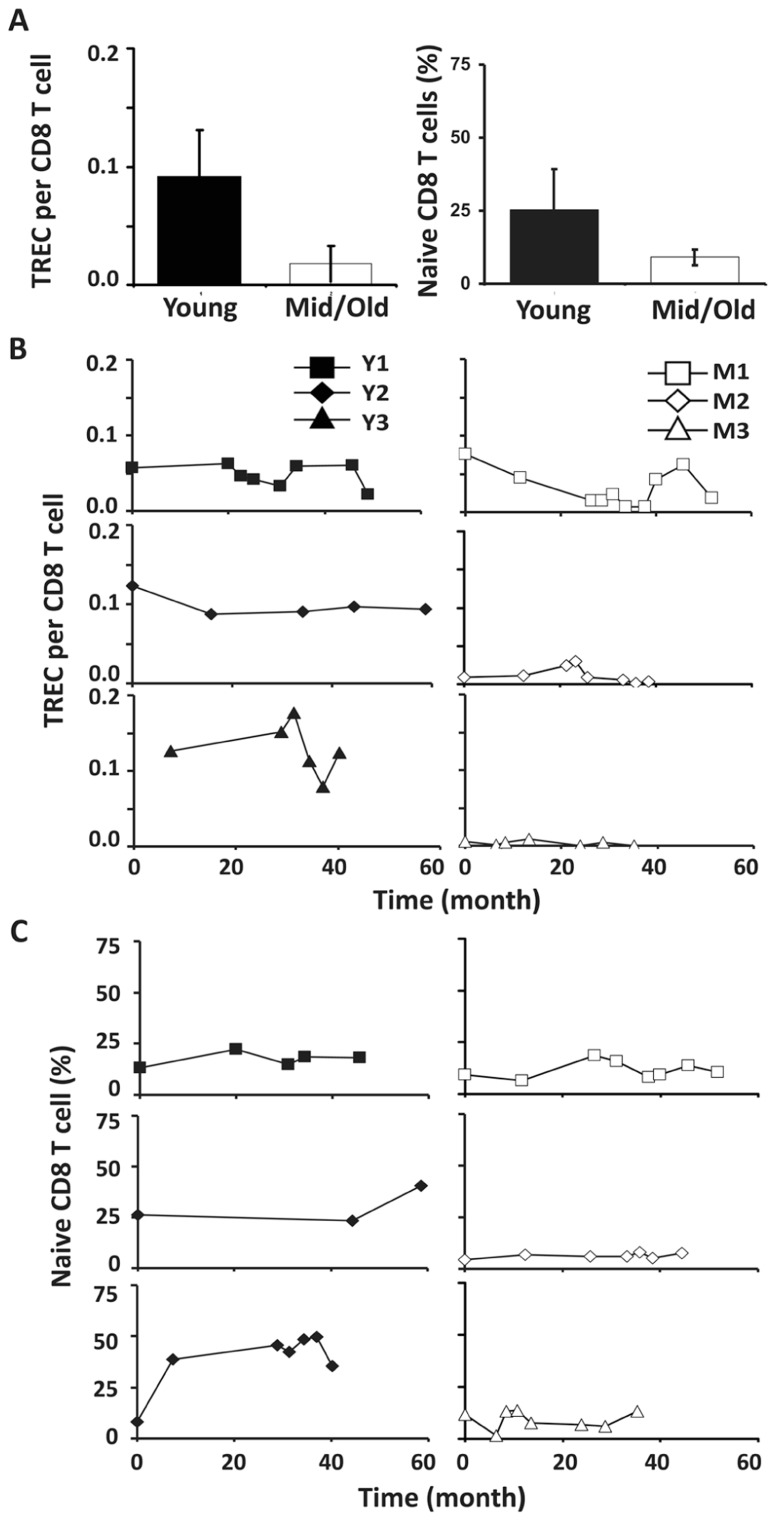
**CD8 TREC levels were variable in both young and middle-aged donors. (A)** TREC counts (left) and the percentages of naïve CD8 T cells (right) are presented (*P* < 0.05). **(B)** TREC counts in CD8 T cells isolated from young (left panels) and middle-aged (right panels) donors are shown. **(C)** naïve (CD45RA^+^ and CD62L^+^ or CCR7^+^) CD8 T cells were examined. PBMC were counterstained with CD8 and CD45RA and CD62L or CCR7 and examined by FACS analysis.

**Figure A3:**
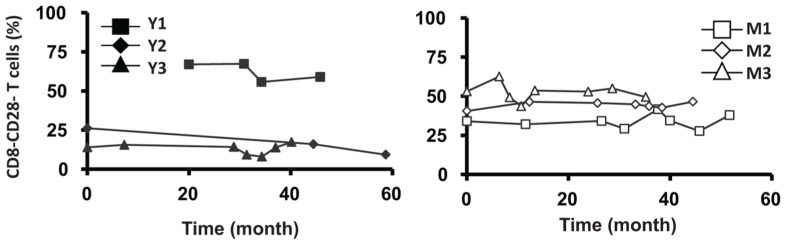
**CD8^+^CD28^-^ T cells of six frequent leukapheresis donors throughout 3–5 years.** PBMC were isolated and stained with CD8 and CD28. The percents of CD8^+^CD28^-^ in total CD8 T cells are presented.

Similar to CD4 T cells, TREC frequencies in CD8 T cells were higher in young than in the middle-aged/old donors (*P* < 0.05; **Figure 4A**). Individually, we observed some fluctuation in TREC counts in both young and middle-aged/old donors (**Figure 4B**). We then analyzed naïve CD8 T cells defined by CD45RA^+^ and CD62L^+^ or CCR7^+^ and observed the difference between young and middle-aged/old adults (**Figure 4C**). As the relationship between CD31 expression and recent thymic emigrants of CD8 T cells has not been defined, we did not observe a close association between TREC counts and the number of naïve CD8 T cells (**Figures 4B,C**). In addition, we analyzed CD28^-^CD8 T cells and found that the percents of CD28^-^CD8 T cells were relatively stable over time in both young and middle-aged/old donors (**Figure A3** in Appendix).

### MORE FLUCTUATIONS OF TCR β VARIABLE GENE USAGE IN YOUNG THAN IN MIDDLE-AGED/OLD DONORS

To ascertain if the usage of another key factor of T cell homeostasis and function changes over time, we examined TCR β variable gene (Vβ) usage in these six donors longitudinally with a panel of antibodies specific for 24 different Vβ genes. We investigated the percent of CD4 and CD8 T cells that stained positive for one of the 24 different Vβ chains contained in our panel of antibodies. The total percentage of CD4 and CD8 T cells covered by these Vβ antibodies was approximately 50% each and similar between young and middle-aged/old donors (**Table 2**). The magnitude of measurable fluctuations of each Vβ change was determined by measuring the same Vβ’s two to three different times from the same donors and the same sample (time point). The SD of these differences was calculated for each Vβ and used as a measure to determine actual changes in Vβ usage over time. To evaluate the change longitudinally, we applied two criteria: (1) the degree of change in Vβ usage must have been greater than the SD calculated for that particular Vβ and (2) the change in Vβ percentage had to be consistent for at least two consecutive time points of each donor. In agreement with the TREC changes, we observed more Vβ usage changes in young donors than in middle-aged/old donors in both CD4 and CD8 T cells (**Table 2**).

**Table 2:** Vβ staining and change in CD4 and CD8T cells overtime.

	CD4			CD8			Time span (month)
Donor	Percent^a^	Increase	Decrease	Percent	Increase	Decrease
Y1	49.7			47.5	14		46
Y2	47.5	3, 13.2*	5.3, 7.1, 1*, 5.1*	42.4	3	5.3, 5.1, 7.2	59
Y3	42.4		2, 17	38.8		8, 14, 21.3	26
M1	38.5		1	34.5		1, 23	25
M2	47.0			38.4		8	32
M3	61.3		11	46.9		5.2*	35

a TCR Vβ repertoire usage was determined using a panel of 24 fluorochorme-conjugated antibodies against individual Vβ epitopes in PBMC along with antibodies against either CD4 or CD8. The sum of the percent of T cells staining positive for each Vβ genes are shown for each donor.

* The SD of measurement error for each Vβ was calculated (see Results). The Vβ gene designation without * indicates the change ≥ 1 SD but < 2 SD, and with * indicates change ≥ 2 SD.

### DIFFERENT CHANGES OF TELOMERE LENGTHS IN YOUNG AND MIDDLE-AGED/OLD LEUKAPHERESIS DONORS

**FIGURE 5:**
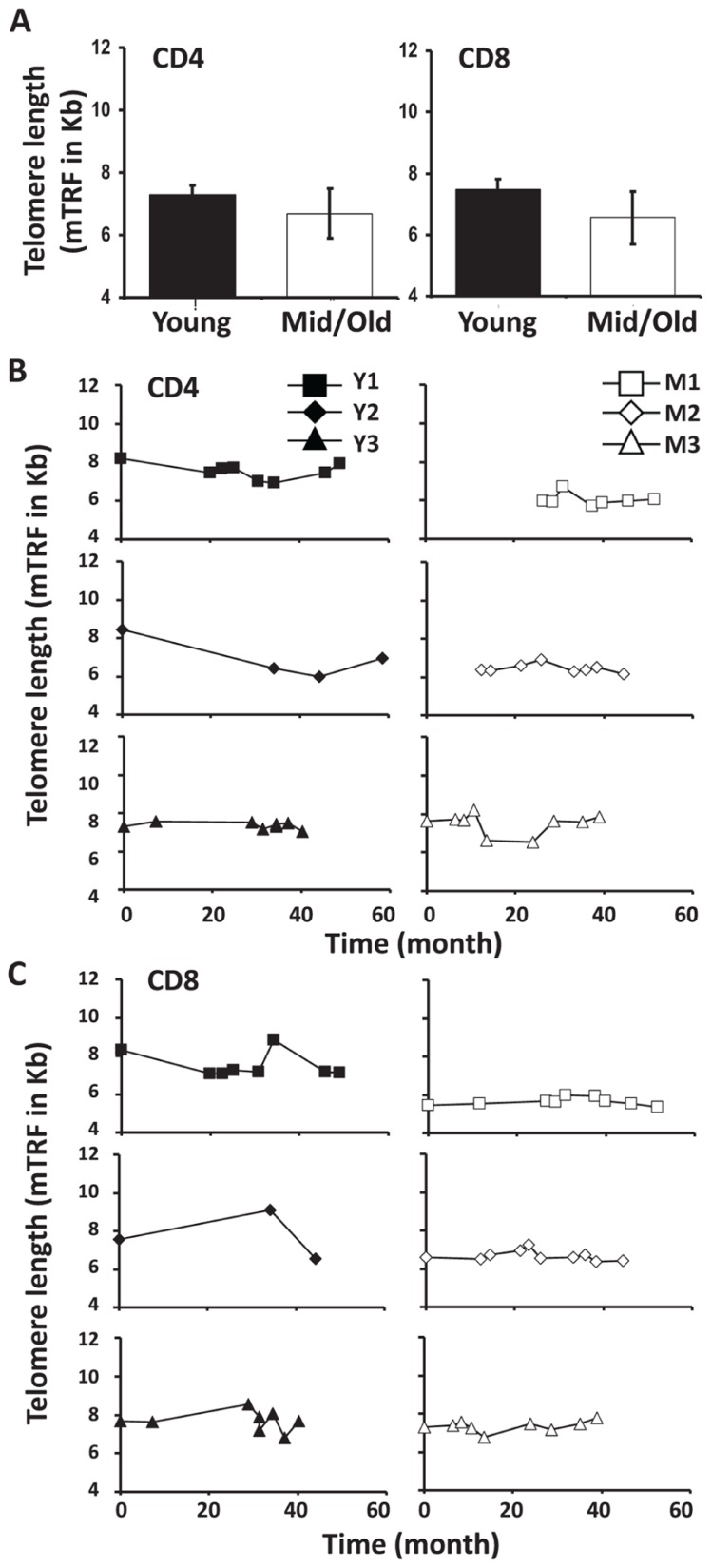
**Telomere attrition was higher in young than in middle-aged adults. (A)** Telomere length of CD4 and CD8 T cells from young and middle-aged donors are presented as mean and SD. **(B)** The CD4 T cell Telomere lengths of each donor are shown, young (left), and middle-aged (right). **(C)** CD8 Telomere lengths of T cells from each donor are shown, young (left), and middle-aged (right).

Telomere attrition has been well documented during cell division and aging. To determine if telomere length changed in CD4 and CD8 T cells of young and middle-aged/old leukapheresis donors, we used a flow-FISH method and measured the telomere lengths of CD4 and CD8 T cells at multiple time points during the 3–5 year donation. Telomeres were measured by mean florescence intensity (MFI) using flow cytometry and then converted from mean florescence intensity to kilobase pairs. Consistent with the above findings, telomere length was more stable in middle-aged/old than in young donors over 3–5 years (**Figure 5B**). The telomere attrition rates are 245 bp/year and 5 bp/year for young and middle-aged/old donors CD4 cells, respectively (**Figure 5B**). The mean telomere attrition rates are 119 bp/year and 22 bp/year for young and middle-aged/old donors CD8 cells, respectively (**Figure 5C**). Compared to the previously reported telomere attrition rates in CD4 and CD8 T cells from cross-sectional analysis, the telomere attrition rates were much higher in young donors and lower in the middle-aged donors. It remains to be determined whether this is a general characteristic of telomere length change in young and middle-aged adults.

## DISCUSSION

The design of this study enabled us to examine T cell regeneration in normal humans *in vivo* without the confounding factors of severe lymphopenia or disease observed in other systems examining human T cell homeostasis (Sachsenberg et al., 1998; McCune et al., 2000; Neese et al., 2002; Douek et al., 2003; Krupica et al., 2006; Williams et al., 2007; Daguindau et al., 2008). As expected, we found that young donors had more TREC and longer telomeres in T cells expressing either CD4 or CD8. Higher frequencies of CD31^+^ naïve CD4 T cells were found in young than in middle-aged/old donors. While the middle-aged/old donors displayed more stability in most measurements, young donors showed a great deal of variation in TREC frequencies, Vβ usage, and telomere lengths over the course of 3–5 years. These findings provide evidence that immune cell homeostasis and TCR repertoire are more dynamic in young than in middle-age/old adults, reflecting a prominent contribution of thymic output in the peripheral pool of T cells. Furthermore, they suggest regeneration of lymphocytes and monocytes from either central progenitor cells or peripheral expansion is robust throughout adult life up to the early 70s. Certainly, further study with more subjects over a longer period is needed not only to verify these findings but also to detect some suttle but substantial changes that only can be observed over a longer period of time.

Leukapheresis removes approximately 10% of PBMC that consists of mainly lymphocytes (T, B, and NK cells) and monocytes. Despite the limited numbers of donors and the relatively short time course (3–5 years) of this study, our longitudinal analysis reveals firsthand information regarding the regeneration lymphocytes and monocytes *in vivo*. In monocyte and T cell compartments, the regeneration was robust in both age groups as manifested by stable cell counts throughout the 3–5 year time course. In CD4 T cell subsets, there are donor differences in the naïve and memory CD4 T cell ratio but no obvious age-related changes in the naïve and memory CD4 T cell ratio. In CD8 T cells, there is no obvious increase of CD28^-^CD8 T cell percentage during the course of leukapheresis. Intriguingly, decreasing B cell percents and increasing NK cell percents were observed in some young donors, implying that regeneration of different types of lymphocytes may be different. However, the precise physiological relevance of these differences will require further study with a larger sample size and possibly a longer period of examination (e.g., 10 or longer years) and control donors not undergoing frequent leukapheresis.

Revealing reliable markers expressed exclusively on newly generated naïve T cells would greatly facilitate the tracking of *in vivo* changes undergone by recent thymic emigrants. CD31 expression has been wildly used as a marker of newly generated naïve T cells. It has recently been reported that newly generated CD31^+^ naïve T cells are capable of retaining CD31 expression after undergoing division *in vivo* (Kilpatrick et al., 2008). In agreement with this observation, we found the percentage of CD31^+^ in the CD4 naïve T cell compartment was more stable than the TREC counts observed in CD4 T cells in young donors over our 3–5 year time course. Therefore, TREC frequencies may serve as a more sensitive marker for newly generated naïve CD4 T cells than the percentage of CD31^+^ CD4 T cells. Compared to CD31^-^ T cells, CD31^+^CD4 T cells may be regulated by IL-7 (Azevedo et al., 2009). Previously we have demonstrated that naïve CD4 T cells respond to IL-7 with faster cell division and better survival than memory CD4 T cells (Yang et al., 2008). This phenomenon is due partly to the ability of naïve CD4 T cells to up-regulate telomerase in response to IL-7 and similarly CD31^+^ T cells have been shown to have high telomerase activity (Junge et al., 2007). Whether telomerase expression can be used as an additional parameter to distinguish newly generated naïve T cells will require further study. Identification and characterization of new markers of naïve T cells will be necessary to enhance our understanding of naïve T cell homeostasis *in vivo*.

The large number of cells made available by leukapheresis provides a unique resource to study immune cell function *ex vivo* in humans. Leukapheresis has also been proposed as a therapy for some autoimmune diseases such as rheumatoid arthritis (Sakai et al., 2009). However, the long-term effect of such therapy is unknown. More studies are needed to better understand immune cell regeneration and homeostasis (lymphoid and myeloid lineages) *in vivo*, and such knowledge will serve as a foundation for developing new therapeutic applications.

## Conflict of Interest Statement

The authors declare that the research was conducted in the absence of any commercial or financial relationships that could be construed as a potential conflict of interest.
